# A Ferrocene-Porphyrin Ligand for Multi-Transduction Chemical Sensor Development

**DOI:** 10.3390/s130505841

**Published:** 2013-05-07

**Authors:** Larisa Lvova, Pierluca Galloni, Barbara Floris, Ingemar Lundström, Roberto Paolesse, Corrado Di Natale

**Affiliations:** 1 Department of Chemical Science and Technologies, University of Rome Tor Vergata, via della Ricerca Scientifica, 1, Rome 00133, Italy; E-Mails: larisa.lvova@uniroma2.it (L.L.); galloni@scienze.uniroma2.it (P.G.); floris@uniroma2.it (B.F.); paolesse@uniroma2.it (R.P.); 2 Faculty of Biology and Soil Science, St. Petersburg State University, Oraniembaum sh., 2, St. Petersburg 198504, Russia; E-Mail: llvova@hotmail.com; 3 IFM, Linköping University, Linköping SE-581 83, Sweden; E-Mail: ingemar@ifm.liu.se; 4 Department of Electronic Engineering, University of Rome Tor Vergata, via Politecnico, 1, Rome 00133, Italy

**Keywords:** porphyrin-ferrocene ligand, opto-potentiometric transduction, transition metals detection

## Abstract

5,10,15,20-Tetraferrocenyl porphyrin, H_2_TFcP, a simple example of a donor-acceptor system, was tested as ligand for the development of a novel multi-transduction chemical sensors aimed at the determination of transition metal ions. The fluorescence energy transfer between ferrocene donor and porphyrin acceptor sub-units was considered. The simultaneously measured optical and potentiometric responses of solvent polymeric membranes based on H_2_TFcP permitted the detection of lead ions in sample solutions, in the concentration range from 2.7 × 10^−7^ to 3.0 × 10^−3^ M. The detection limit of lead determination was 0.27 μM, low enough to perform the direct analysis of Pb^2+^ in natural waters.

## 1. Introduction

Multi-metal contamination has become nowadays a significant problem in many countries and whole regions [1–5]. The growing industrial impact, the accumulation of factory and domestic wastes and incorrect preservation are the main causes of the widespread environmental pollution by transition and heavy metals. The high content of transition metals in soil, air and drinking water cause their consecutive accumulation in humans and this phenomenon has been connected to the insurgence of allergies, tumors, and other serious diseases, such as genetic pathologies, especially in children [6]. The necessity for careful monitoring and frequent analysis of transition metals content in the environment, and in natural and drinking water in particular, is thus self-evident.

Among the standard methods of transition metal analysis, Atomic Absorption Spectrometry, Inductively Coupled Plasma Atomic Emission Spectrometry, Inductively Coupled Plasma Mass Spectrometry are the most commonly used techniques [7]. All the abovementioned methods require careful sample pretreatment prior to analysis, and costly and sophisticated equipment, which requires the involvement of experienced personnel. The application of chemical sensors has found a lot of interest for environmental monitoring tasks, and for transition and heavy metal ions detection in particular [8], due to the obvious advantages of simple preparation and handling, low cost, reasonable selectivity and improved sensitivity.

In chemical sensors the sensing event consists of the interaction between the sensing material, often containing a specific ligand, and the target analyte, with a further transformation of the obtained information, which can be registered with different (one or more) transduction principles, into a useful analytical signal. Many specific ligands containing oxygen, sulfur or/and nitrogen atoms in their structures in order to have high transition metal coordination ability, were previously reported for chemical sensor development [9–18].

Among them porphyrins have attracted particular interest due to their rich red-ox chemistry, delocalized aromatic π-system and optical absorption that extends over almost all visible region, which gives the possibility to apply different transduction principles (electrochemical, optical, mechanical, *etc.*) for the signal detection of porphyrin-based sensors [19]. Previously we have reported the application of metalloporphyrin-based chemical sensors for opto-electrochemical dual mode discrimination of vegetable oils [20] and for the detection of hazardous food additives of the Sudan family [21,22]. The free-base porphyrins were demonstrated to form sitting-atop complexes with various transition metal ions [23] and previously transition-metal sensitive potentiometric sensors based on free-base porphyrin ligands were reported [24,25]. The strength of such an interaction can be tuned by the proper choice of porphyrin ring side-substituents that may act as the secondary chelating centers of a target ion, or/and donate/withdraw the additional electronic density to/from the porphyrin core.

Ferrocene-substituted porphyrins represent covalently linked donor-acceptor systems that can undergo photo-initiated electron transfer in which the porphyrin may accept electrons from the ferrocene substituent/s that have relatively low oxidation potential. The synthesis and the investigation of the porphyrin-ferrocenes physico-chemical properties have received lots of attention in the last decade [26,27]. Porphyrin-ferrocenes were actively applied in photovoltaic light harvesting and energy conversion systems [28–30], and intelligent surface coatings [31,32].

On the contrary, only few examples of porphyrin-ferrocene-based chemosensors can be found in the literature. Thus, Bucher *et al.* have reported in [33] the possibility of the detection of amine-containing species, by means of their coordination on the metal center of ferrocene-substituted Zh(II)porphyrin and further ligand self-assembly and supramolecular dimer formation. As a result of the efficient electronic communication between the π-systems of the porphyrin and ferrocene moieties, coordination of a Lewis base to the zinc porphyrin brings about characteristic shifts in the redox potential of the ferrocene, enabling the electrochemical sensing of neutral ligands, such as pyridine, imidazole and 2-methylimidazole. The substitution of the ferrocene moiety in the Zn-porphyrin-ferrocene ligand discussed above with an alkylammonium group gave the corresponding cationic zinc porphyrin species, able to act as an effective ditopic anion binding through the metallic zinc center and the quaternary ammonium group [34]. The binding of several anions, such as NO_3_^−^, HSO_4_^−^, H_2_PO_4_^−^, Cl^−^, Br^−^ and F^−^, resulted in anion-dependent changes of the redox potentials of both the ferrocenyl and porphyrin moiety, thus allowing selective anion sensing. Previously the similar principle was reported by Beer *et al.* for ferrocene-zinc porphyrin and porphyrin-cobaltocenium receptors, where the selective anion complexation resulted in cathodic electrochemical perturbations of both the porphyrin and ferrocene/cobaltocenium redox processes [35,36]. To the best of our knowledge, no applications of ferrocene-porphyrins for cations sensing were reported up to now.

**Figure 1.**
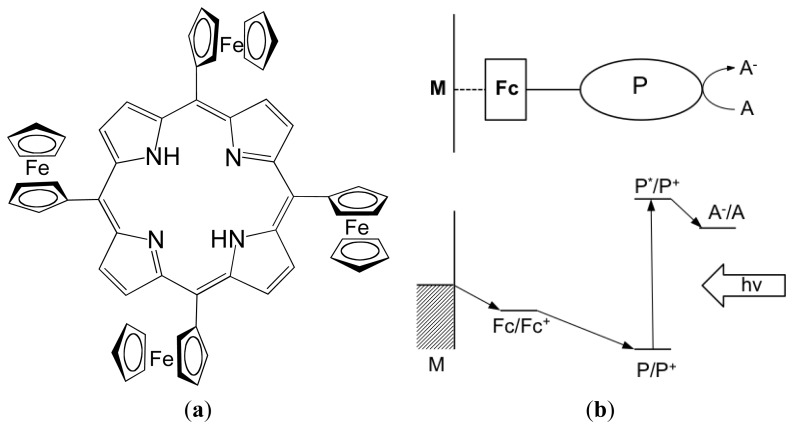
(**a**) Chemical structure of free-base meso-tetraferrocene porphyrin, H_2_TFcP; (**b**) energy level diagram showing possible photochemical events in porphyrin-ferrocene in contact with a conducting transducer M and solution containing an electron acceptor A.

In this work we study the free-base meso-tetra-ferrocenyl porphyrin conjugate, H_2_TFcP, as a novel sensing material for the development of multi-transduction opto-electrochemical sensors for transition metal detection. The molecular structure of H_2_TFcP is shown in Figure 1(a). It was previously demonstrated in [29], that the conjugate formed by a photoactive porphyrin group (P) and an electron donor ferrocene group (Fc) in contact with a solution containing an electron acceptor (A), whose redox potential is more negative than that of ferrocene, is a good candidate for a stable and efficient photoconversion device with uphill electron transfer (Figure 1(b)). Following this concept, our idea was to measure the hyphenated opto-electrochemical response of porphyrin-ferrocene conjugate based sensor, induced by the interaction of light-illuminated porphyrin group with solutions containing transition metal ions. The potentiometric response of H_2_TFcP-based sensing films towards various metal ions was studied, together with the film luminescence intensity evaluated by means of the Computer Screen Photoassisted Technique (CSPT), which applies familiar devices, such as LCD computer screens and web-cameras, as light sources and signal detectors, respectively [37,38]. The enhanced sensitivity of H_2_TFcP-based films towards copper, and especially lead ions, and the significant improvement of detection limits achieved by the multi-transduction approach application to the same sensing film are presented below.

## 2. Experimental Section

### 2.1. Reagents

High molecular weight poly(vinyl chloride) (PVC), bis(2-ethylhexyl) sebacate (DOS) plasticizer, potassium tetrakis-(4-chlorophenyl)borate (TpClPBK) anionic additive and tetrabutylammonium perchlorate (TBAClO_4_) supporting electrolyte salt, 2-amino-2-(hydroxymethyl)-1,3-propanediol (TRIS) buffering agent were purchased from Fluka (Milan, Italy). Tetrahydrofuran (THF), dimethylformamide (DMF), acetonitrile (ACN) and dichloromethane solvents were purchased from Sigma-Aldrich (St. Louis, MO, USA). THF was freshly distilled prior to use. Millipore grade water was used for aqueous solution preparation. All the other chemicals were of analytical grade and used without further purification. H_2_TFcP was prepared according to a previously reported synthetic procedure [26]. The electrochemical properties of H_2_TFcP were previously studied in detail in [31].

### 2.2. H_2_TFcP Fluorescence Study

Fluorescence spectroscopy measurements were performed with Shimadzu RF-1501 (Kyoto, Japan) spectrophotometer in a glass cell with 1 cm path length. The H_2_TFcP solutions of initial Soret absorbance intensity of 0.1 units (about 3.5 × 10^−5^ M of H_2_TFcP) were prepared in tree different solvents, namely DMF, ACN and CH_2_Cl_2_. The consecutive injections of two stock solutions of Cu^2+^ and Pb^2+^-nitrates were then performed into H_2_TFcP in DMF. DMF was chosen since this solvent is mixable with waster and permitted the direct preparation of DMF-aqueous solutions with the final concentration of target metal ions in the range from 1.7 × 10^−6^ M to 2.2 × 10^−3^ M. After the excitation at 430 nm the solution emission spectra were measured in 600–750 nm range with maximum of fluorescence intensity registered at about 650 nm.

### 2.3. Cyclic Voltammetry of H_2_TFcP Solutions

The CV experiments on H_2_TFcP in DMF and CH_2_Cl_2_ solvents were performed. The CVs were taken in −0.5 to 1 V range with 100 mV/s scan rate in 1 mM solutions of H_2_TFcP containing 0.1 M of TBAClO_4_ supporting electrolyte salt in a standard 3 electrode cell with ITO working, SCE reference and Pt wire counter electrodes. The AMEL 7050 (AMEL Instruments, Milan, Italy) potentiostat was applied for CV characterizations.

### 2.4. Solvent Polymeric Membranes Preparation

Polymeric membranes of 100 mg weight were prepared by incorporation of 1wt% of H_2_TFcP ligand in DOS-plasticized PVC matrix (1:2 ratio in weight) and determined amount of lipophilic TpClPBK salt additive. In particular, three polymeric membranes of following compositions were prepared: **Mb1**: PVC/DOS/H_2_TFcP; **Mb2**: PVC/DOS/H_2_TFcP/TpClPBK(0.25wt%); **Mb3**: PVC/DOS/H_2_TFcP/TpClPBK(0.4 wt%). All membrane components were dissolved in 1 mL of THF and about 15 μL of each membrane cocktail were then cast onto 1.5 cm length, 7 mm width conductive ITO glass slides (nominal resistance of 30–60 W/cm^2^, Aldrich). Solvent was allowed to evaporate overnight to form a polymeric membrane films well adhesive to the glass slide surface. The film thickness was evaluated of about 250 μm.

### 2.5. Potentiometric and CSPT Measurements with H_2_TFcP-Based Polymeric Membranes

The potentiometric response of H_2_TFc-based polymeric membranes was evaluated in the individual solutions of several inorganic salts in the range from 2.7 × 10^−7^ M to 1.0 × 10^−3^ M at distilled water or TRIS-HNO_3_ pH = 7.4 buffer background at ambient temperature (+22 °C). Prior the potentiometric testing the freshly prepared films were soaked in 0.01M NaCl aqueous solution for at least 24 hours.

The potentials of ITO glass slides modified with H_2_TFcP-based membranes were measured *versus* a SCE reference electrode (AMEL Instruments, Milan, Italy) applying LiquiLab (ECOSENS srl, Rome, Italy) high-impedance analog-to-digital potentiometer. The effect of pH on membrane potentiometric responses has been evaluated in universal buffer solution (UBS, prepared with 6.7 mM citric acid, 11.4 mM boric acid, and 0.01 M NaH_2_PO4, initial pH 2.8) by additions of 1 M NaOH, to the final pH 10.5. A glass pH electrode (AMEL, Instruments, Milan, Italy, model 411CGG/6) was used to control the solution pH.

In CSPT measurements the unique ITO glass slide with three deposited spots of **Mb1**–**Mb3** polymeric films was placed in the transparent cuvette and backside illuminated with the polychromic light provided by a TFT-LCD (Samsung, Seoul, Korea) computer monitor screen. The outcoming optical signal was captured with a digital web-cam (Logitech Quickcam® for Notebooks, 352 × 288 pixels resolution). The H_2_TFcP-based films optical intensity variation upon the exposure to the growing concentrations of target metal ions and illumination by the sequence of 50 colors was registered and transformed in analytically useful signal by in-house written MATLAB codes (v.7.9, 2010b, The MathWorks, Inc., Natick, MA, USA). The measurement cell was properly shielded from ambient illumination. In order to assure the uniformity of the measured signal, the mean optical intensity of the representative region (spot) on film of the defined area (five pixels in this study) was measured. For this the optical intensities of the pixels inside each spot for every film were averaged and after subtraction of the background (the region of image without film), contribute to form the CSPT fingerprint. Red, green and blue channel (RGB) signals are concatenated in this order and a fingerprint vector containing 150 elements (composed of 50 illumination colors measured for three camera channels) is formed for each film. The sequence of these 150 elements was called “illumination index”. For each of 150 points of “illumination index” of RGB fingerprint the corresponding webcam optical intensity was measured and plotted along Y-axis. The optical intensity is a relative value that is determined by the film luminescence, and this value varies under film exposure to different analyte amounts. In the present study the registered optical intensity varied in the range from 60 to 250 units. The decrease of the optical intensity corresponds to the situation when the H_2_TFcP carrier ligates to the analyte and the overall amount of free ligand in membrane decreases. In some particular cases the increase of membrane luminescence (and, as a consequence of registered optical intensity) under lead ions exposure was registered. Such a situation was assumed to the formation of analyte-ligands aggregates of complex geometry, thus making the film “more transparent”, and hence, more luminescent (see Section 3.3 of Results and Discussion for more details).

Moreover, the opto-potentiometric response of the same H_2_TFc-based polymeric membranes was evaluated. For this, the separate electrical channels for each of **Mb1**–**Mb3** polymeric film spots were cut out on a unique ITO glass slide; this glass, together with a thin double-junction SCE reference electrode, were placed in a transparent cuvette containing background solution. Upon the addition of analyte the measured cell was illuminated by polychromic light as above described and optical (in form of videos registered by web-cam) and potentiometric responses of the same films were registered simultaneously.

### 2.6. Multivariate Data Treatment

Principal component analysis (PCA) was applied for interpretation of the CSPT-potentiometric response of H_2_TFcP-based membranes. The Partial Least Squares Regression (PLS) method was applied in order to obtain the calibration curve of the hyphenated CSPT-potentiometric response of H_2_TFcP-based sensors to the amounts of target transition metal analytes in standard calibration solutions of known concentrations.

The PLS regression method is a multivariate method, that relates the variations in one or several analyte variables (Y-variables) to the variations of several sensor response predictors (X-variables), with explanatory or predictive purposes. The goal of PLS regression is to predict *Y* from *X* and to describe their common structure. For this the matrix denoted *Y* = *M*·*K*, that consists of *M* observations described by *K* variables, is correlated to the matrix *X* = *M*·*J* consisting of *J* predictors collected on these *M* observations. When, as in our case, *Y* is a vector (formed by different concentrations of target ion in analyzed samples) and *X* is full rank (the matrix of optical and potentiometric responses of **Mb1**–**Mb3**), the prediction goal is accomplished using ordinary multiple regression, that implies least squares method supposing that the analyte content can be represented as a linear combination of individual sensor responses: Y = β_1_ · x_1_ + … + β_n_ · x_n_ + *e*, where *β_i_* is a coefficient influencing to the signal *Y*, *x_j_* is the signal obtained only for sensor *j*, *e* is the residual error. In this equation *Y* and *x_j_* are known values, while *β* can be evaluated by least square method.

Prior to analysis the mean normalization of data was performed that allows us to simultaneously treat the potentiometric and optical CSPT response of sensors. The validation of the PLS model was performed using the cross-validation approach. The Root Mean Square Error of Prediction and Validation (RMSEP and RMSEV, respectively), the slopes and the correlation coefficients of predicted *versus* measured correlation line were used to evaluate the efficiency of developed sensors. Data treatment was performed with Unscrambler software (v.9.1, 2004, CAMO Software AS, Oslo, Norway).

## 3. Results and Discussion

### 3.1. Potentiometric Properties of H_2_TFcP Based Polymeric Membranes

The free-base porphyrin is a tetradentate dianionic ligand, where the coordination of a metal ion involves the deprotonation of two N-pyrrolic atoms and the successive out-of-plane ion binding with the formation of the so called sitting-atop complex. From this point of view the porphyrin-ferrocene conjugates, where an additional electronic density can be provided to the porphyrin π-system by the ferrocene substitutes, can be considered a good and promising ligating agent for transition metal ions. Based on this suggestion, we have first studied the potentiometric responses of H_2_TFcP based membranes **Mb1**–**Mb3** towards a series of different metal salts in the 2.7 × 10^−7^ M to 5.0 × 10^−2^ M concentration range in distilled water or 0.01 M TRIS-HNO_3_ pH 7.4 buffer background. The amount of 1wt% of H_2_TFcP incorporated in the membranes was determined by the ligand solubility in the membrane phase. We have initially supposed a neutral cationic carrier working mechanism for the H_2_TFcP ligand. It was previously demonstrated that the addition of anionic lipophilic sites significantly improves the selective properties of membranes based on neutral cationic carriers, facilitating the target cation transport in the membrane phase, tuning the ion-exchange properties and decreasing significantly the overall membrane resistance [39].

In order to check the possible stoichiometry of ligand-metal complexes formed during the sensing event in the polymeric membrane phase, the properties of **Mb1** without any lipophilic additive were compared to **Mb2** and **Mb3**, both containing anionic lipophilic TpClPB^−^ sites of 0.2 and 0.4wt% respectively. The slopes of potentiometric calibration curves of **Mb1** and **Mb2** in all tested salts are given in Table 1. The potentiometric behavior of **Mb3** was clearly determined by the high amount of TpClPBK, and the selectivity pattern typical of cation-exchanger-based membranes governed by the hydration energies of the tested cations was observed (data not shown). As can be seen from Table 1, NH_4_^+^, alkali- (Na^+^, K^+^, Li^+^), alkali-earth (Ca^2+^, Mg^2+^), Co^2+^ and Cd^2+^-ions did not show any significant influence on the **Mb1** and **Mb2** potentiometric responses. On the contrary, linear responses in E (mV)-pX coordinates, with the slopes of +17.0 and +23.4 mV/pCu and +15.1 and +30.8 mV/pPb, were registered for **Mb1** and **Mb2** respectively. The **Mb2** demonstrated close to the theoretical Nernstian slopes both towards Cu^2+^ and Pb^2+^ ions, in comparison to the lower slopes of **Mb1**, due to the 0.2wt% TpClPBK addition. We suppose that the presence of lipophilic anionic TpClPB^−^ sites induces the flux of target cations in membrane phase, where ligand-analyte aggregates of complex geometry are formed (see the optical response section for more details).

Moreover, the smaller pH influence on **Mb2** cationic response in compatison to the **Mb1** prepared without cation-exchenger was registered (slope of 13.2 ± 2.1 mV/pH for **Mb2** in respect to 39.7 ± 1.8 mV/pH for **Mb1**). In TRIS-HNO_3_ background solution with a fixed pH 7.4 the higher slopes towards lead (+31.5 and +34.8 mV/pPb) and copper (+35.2 and +36.8 mV/pCu) ions were obtained for **Mb1** and **Mb2** correspondingly, causing at the same time the deterioration of the selective response towards Pb^2+^- over the Cu^2+^-ions.

Unfortunately, the potentiometric working range of **Mb2** towards both copper and lead ions was quite narrow and lay in the concentration interval from 1.1 × 10^−4^ M to 5.0 × 10^−2^ M of target analyte. Such a high detection limit is a serious drawback for any potential H_2_TFcP-based sensor applications for environmental monitoring and, in particular, for drinking water analysis where, according to the international regulations, the amount of lead and copper should not excees 0.25 and 8.85 μM correspondingly [7]. Hence, the direct potentiometric detection of transition metals content in natural and potable waters by means of H_2_TFcP-based membranes results difficult without preliminary probe preconcentration.

In our previous studies it was shown that the application of multi-transduction approach to the same sensing material may significantly improve the resulting sensor performance [40], and in particular to overcome an unsatisfactory detection limit. For this reason during the second stage of our study we first have evaluated the optical properties of H_2_TFcP ligand and H_2_TFcP-based membranes, and then have applied the simultaneous opto-potentiometric signal acquisition for the evaluations of various metal contents in the aqueous model solutions.

### 3.2. The Fluorescence and CV Evaluations of H_2_TFcP Interaction with Transition Metal Ions

As mentioned above, the properties of directly linked porphyrin-ferrocenes were previously studied, showing a significant charge separation and recombination inside the same molecule, with the presence of several transduction energetic states, which indicated the possibility for such molecules to work as light-sensitive ligands [41,42]. It was also shown that ferrocene quenches porphyrin fluorescence significantly, due to the photo-induced electron transfer from ferrocene to porphyrin [43]. In fact, the quantum yields of porphyrin-ferrocenes are a tenth to a hundred times lower than the tetraphenylporphyrin reference and they decrease with the increase of ferrocene substituents [26,27,44]. For this reason, relatively few studies have been devoted to luminescent electroactive systems including porphyrin-ferrocene conjugates. Schmidt *et al.* examined free-base porphyrins and metalloporphyrins substituted by four ferrocenyl groups, and reported that the fluorescence of the free-base porphyrins increased when the ferrocene pendant groups were oxidized to ferrocenium ions [45].

**Figure 2.**
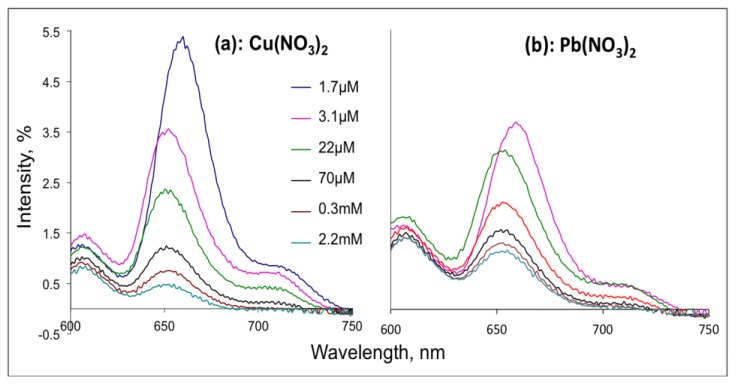
The fluorescence quenching of H_2_TFcP by addition of growing concentrations of (**a**) Cu^2+^ and (**b**) Pb^2+^ ions.

In agreement to the literature data, we observed a Soret absorption band at 434 nm and two enlarged lower energy Q-bands influenced by side ferrocene substituents at 661 and 726 nm in the UV-visible spectrum of H_2_TFcP in CH_2_Cl_2_ [26]. After excitation (430 nm) of H_2_TFcP in CH_2_Cl_2_, DMF or ACN solutions, very moderate emission bands (10% relative quantum yield) at 650 and 720 nm were registered in aprotic CH_2_Cl_2_ and DMF solvents, while in ACN an almost complete fluorescent quenching was found. The low fluorescence quantum yield is a result of the electron donation from Fc to excited porphyrin ring and an almost immediate charge recombination of the solvated porphyrin-ferrocene conjugate. A further fluorescence quenching was observed upon Pb^2+^ or Cu^2+^ cations introduction in a concentration range of 1.67 μM to 2.2 mM, Figure 2, as expected for the heavy-atom effect.

**Figure 3.**
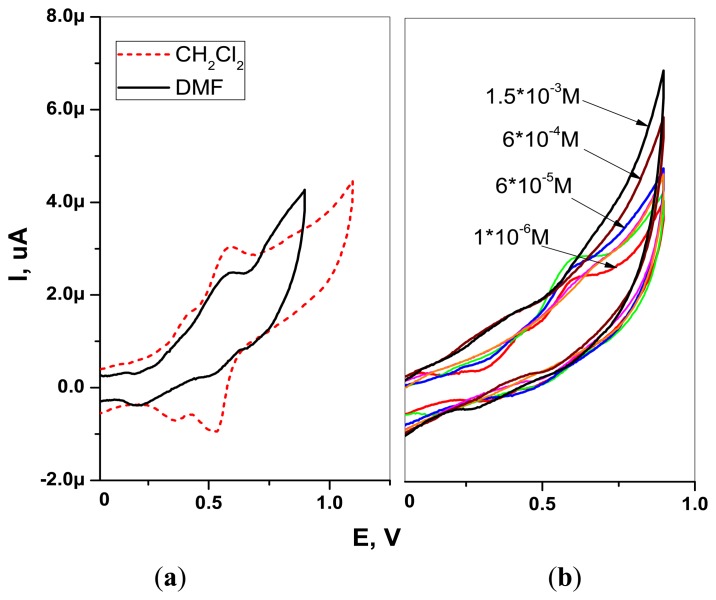
CVs of H_2_TFcP in: **(a)** CH_2_Cl_2_ and DMF solvents; (**b**) in aqueous solutions upon exposure to growing concentrations of Pb^2+^-ions.

The CVs of H_2_TFcP recorded in −0.1 to +1.1 V range showed the presence of a reversible process at +0.55 V, corresponding to the simultaneous oxidation of all the four ferrocene substituents (Fc/Fc+ couple), both in CH_2_Cl_2_ and DMF solvents [46] (Figure 3(a)). The oxidation wave at +0.81 V and +0.85 V, in DMF and CH_2_Cl_2_ respectively, was attributed to porphyrin macrocycle oxidation, in accordance to the previously reported electrochemical studies of porphyrin-ferrocenes [26,44]. This E_1/2_ value is higher than the Cu^2+^ and Pb^2+^ oxidation potentials in aqueous solutions and indicates a potential utility of H_2_TFcP for ligation of such a cations. In fact, cyclic voltammetry of H_2_TFcP in DMF revealed that the addition of growing amounts of Pb^2+^cations results in the growth of the system anodic current, due to the porphyrin ring oxidation and simultaneous Fc/Fc+ peak decrease.

### 3.3. Optical CSPT Response of H_2_TFcP Based Polymeric Membranes

The previously reported CSPT-potentiometric analytical system [20] was applied for the evaluation of **Mb1**–**Mb3** optical response. The development of potentially optical-active sensing materials has determined the choice of the plasticizer used to prepare H_2_TFcP polymeric membranes: among two most common plasticizerz, DOS and oNPOE (*o*-nitrophenyl octyl ether), the former was chosen, due to the well known luminescence quenching activity shown by organic nitro compounds [47,48].

**Figure 4.**
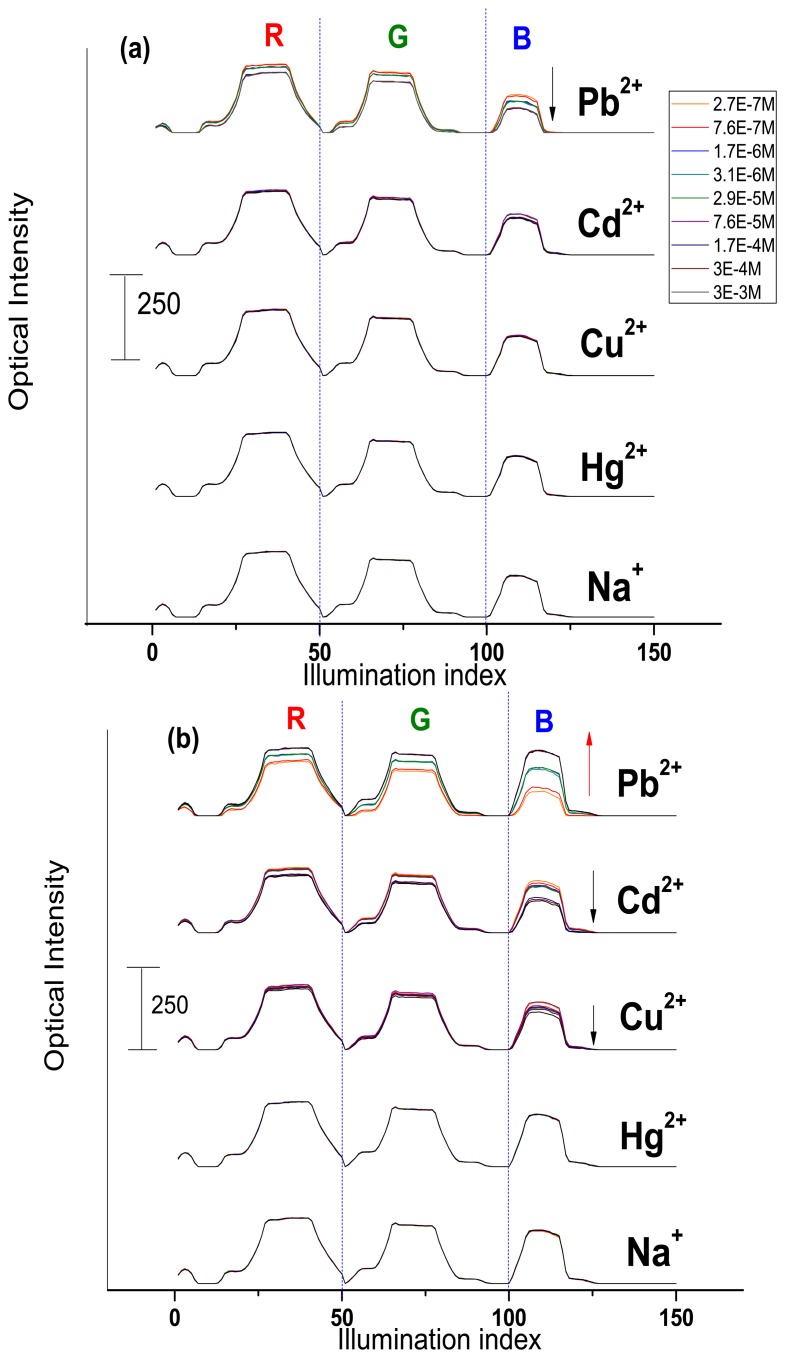

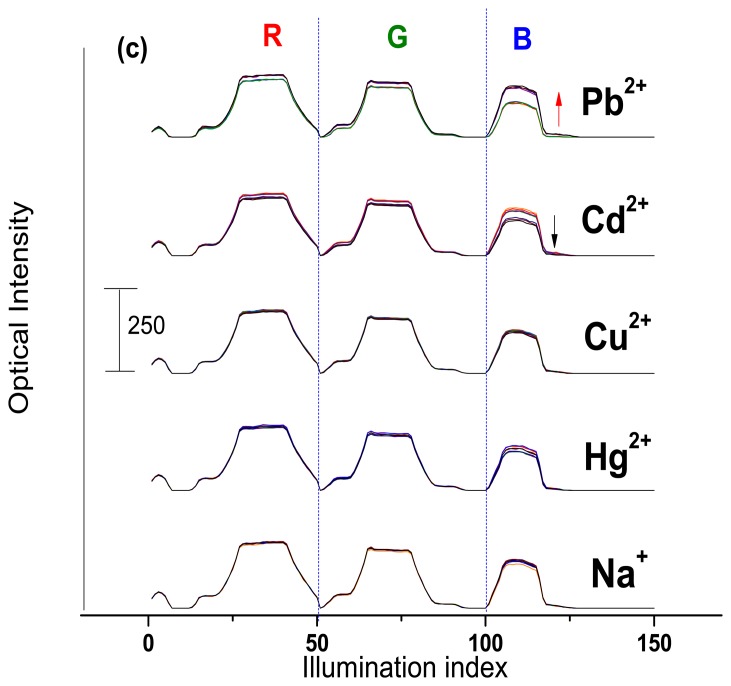
CSPT response of solvent polymeric membranes to several metal ions: (**a**) **Mb1**: PVC/DOS/H_2_TFcP 1wt%; (**b**) **Mb2**: PVC/DOS/H_2_TFcP 1wt%/ TpClPBK 0.25wt%; (**c**) **Mb3**: PVC/DOS/H_2_TFcP 1wt%/ TpClPBK 0.4wt%.

The optical CSPT response towards Na^+^, Cd^2+^, Hg^2+^, Cu^2+^ and Pb^2+^ ion-containing solutions was tested. The sensor response signature was extracted from the webcam video-registrations (see Experimental section for details) and plotted in accordance to the concentration change of the added cations, Figure 4. The measured optical intensity varied in the range from 60 to a maximum of 250 units.

As it can be seen from the Figure 4(a), **Mb1** demonstrated a moderate response towards Pb^2+^ (about 15% of maximum optical intensity value in all RGB interval) and Cd^2+^ (8% of maximum optical intensity value mainly in blue light illumination region) ions concentration change and almost no influence of the other cations was registered. The **Mb1** luminescence in contact with the analyte solution and under polychromic light illumination decreases. This behavior is similar to the above discussed fluorescence measurements of H_2_TFCP/DMF solutions.

The **Mb2**, having the optimal membrane composition found during potentiometric evaluations, demonstrated the CSPT response of higher amplitude to the following decreasing order: Pb^2+^ > Cd^2+^ > Cu^2+^ (Figure 4(b)). The optical intensity variation was about 77% for lead, 27% for cadmium and 16% towards copper ions. Interestingly, the increase of **Mb2** luminescence was observed in Pb^2+^-containing solutions. This luminescence activation is especially high in the blue region of illumination. This confirms our recent considerations on TpClPB^−^ anionic sites incorporation effect on the membrane sensitivity. Evidently, the cation-exchanger presence induces a flux of the target ions in the membrane phase, where the formation of complex geometry H_2_TFcP-Pb^2+^ aggregates results in the decrease of the optical density of the membrane, especially in the blue region that corresponds to the porphyrin Soret band, probably due to the bathochromic effect of the macrocycle aggregation. It is important to notice here that the optic response of **Mb1**, **Mb2** was not influenced neither by alkaline Na^+^ ion no by Hg^2+^. The latter was shown to be a serious interference in the spectroscopic determination of transition metal ions [8].

The further addition of TpClPB^−^, in amount of 0.4wt% in **Mb3**, deteriorates the membrane optical response, which has the smallest optical intensity changes under exposure to lead (about 23% of maximum optical intensity value), and at the same time some interferences of Cd^2+^, Hg^2+^ and Na^+^ (of 23%, 16% and 5% of maximum optical intensity value respectively) on the **Mb3** response was detected, Figure 4(c).

**Figure 5.**
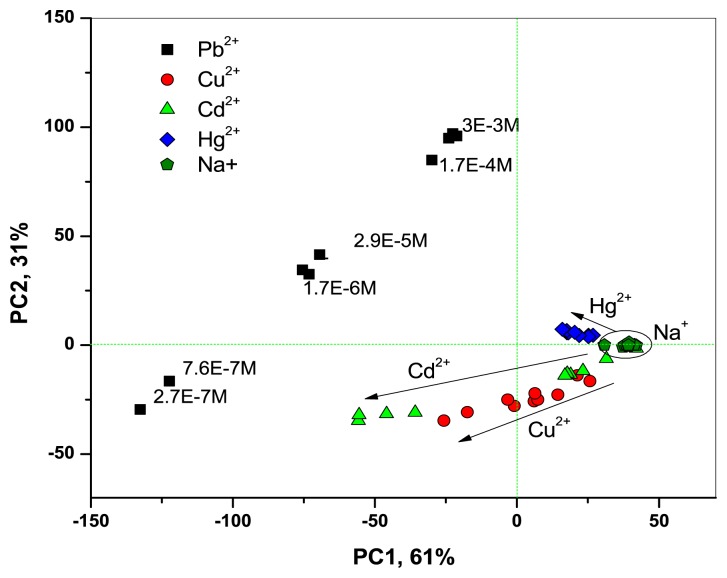
PCA analysis of metal ions with opto-potentiometric **Mb1**–**Mb3** based on H_2_TFcP.

Thanks to the improved optical CSPT-response of **Mb1**–**Mb3**, the fusion of CSPT and potentiometric data permitted us to clearly discriminate between all the tested metal ion species and to observe their concentration gradients, as it is shown in PCA score plot in Figure 5.

The PLS regression technique was utilized to obtain the correlation between CSPT-potentiometric response of the H_2_TFCP-based sensors and lead ion content in calibration solutions of known concentrations. The fusion of potentiometric and optical responses of all three membranes **Mb1**–**Mb3** studied in this work has permitted us to obtain the linear response towards lead in all the tested concentration range from 2.7 × 10^−7^ to 3.0 × 10^−3^ M. The correlation coefficient of the obtained calibration curve was R^2^ = 0.983, and RMSEC = 3.9·μM. The detection limit of lead determination was 0.27 μM, and it was low enough to perform the direct analysis of Pb^2+^ in natural waters.

## 4. Conclusions

It was demonstrated that the simultaneous detection of the potentiometric and optical responses of H_2_TFcP-based solvent polymeric membrane sensors with the following mathematical treatment of the fused data afforded a serious improvement in the transition metal ions detection. The developed sensors give the possibility to perform rapid and inexpensive controls for both quality assessment and quantitative analysis tasks. Further work on the application of H_2_TFcP CSPT-potentiometric sensors for the analysis of real matrices, such as surface and potable waters and soil extracts, are now in progress in our laboratories.

## Figures and Tables

**Table 1.** Potentiometric response slopes of the H_2_TFcP-based membranes towards several analytes in individual solutions.

**Salt**	**Slope (mV/pX a)**	

	**Mb1: H_2_TFcP 1wt%**	**Mb2: H_2_TFcP 1wt%, TpClPBK 0.25wt%**
NaCl	1.0 ± 2.3	1.9 ± 1.3
KCl	1.1 ± 0.9	8.1 ± 5.5
LiCl	−0.7 ± 0.15	0.9 ± 3.0
NH_4_Cl	0.8 ± 0.3	7.3 ± 4.6
MgCl_2_	1.7 ± 0.3	1.8 ± 0.6
Zn(NO_3_)_2_	5.2 ± 0.6	6.0 ± 1.7
CdCl_2_	3.1 ± 0.6	3.0 ± 1.7
CoCl_2_	4.34 ± 0.8	3.2 ± 1.6
Pb(NO_3_)_2_	17.0 ± 5.1	23.4 ± 1.0
Cu(NO_3_)_2_	15.1 ± 1.8	30.8 ± 2.5
NaCl - TRIS	1.3 ± 0.6	11.7 ± 2.7
CdCl_2_ - TRIS	5.3 ± 3.9	6.5 ± 1.8
CoCl_2_ -TRIS	1.7 ± 0.8	1.3 ± 0.3
Pb(NO_3_)_2_ - TRIS	31.5 ± 2.2	34.8 ± 2.0
Cu(NO_3_)_2_ - TRIS	35.2 ± 1.5	36.8 ± 4.9

pH b	39.7 ± 1.8	13.2 ± 2.1

a the mean value for three replicated measurements;

b from 5.5 to 10.2 pH units range.
